# Assessment of Survival Model Performance Following Inclusion of Epstein-Barr Virus DNA Status in Conventional TNM Staging Groups in Epstein-Barr Virus–Related Nasopharyngeal Carcinoma

**DOI:** 10.1001/jamanetworkopen.2021.24721

**Published:** 2021-09-23

**Authors:** Wang-Zhong Li, Hai-Jun Wu, Shu-Hui Lv, Xue-Feng Hu, Hu Liang, Guo-Ying Liu, Nian Lu, Wei-Xin Bei, Xing Lv, Xiang Guo, Wei-Xiong Xia, Yan-Qun Xiang

**Affiliations:** 1 State Key Laboratory of Oncology in South China, Collaborative Innovation Center for Cancer Medicine, Guangdong Key Laboratory of Nasopharyngeal Carcinoma Diagnosis and Therapy, Sun Yat-Sen University Cancer Center, Guangzhou, China; 2 Department of Nasopharyngeal Carcinoma, Sun Yat-Sen University Cancer Center, Guangzhou, China; 3 Department of Radiation Oncology, First People's Hospital of Foshan, Foshan, China; 4 Medical Affairs Office, Fifth Affiliated Hospital of Sun Yat-Sen University, Zhuhai, China

## Abstract

**Importance:**

Nonanatomic prognostic factors complement the traditional anatomic staging system and could be incorporated into the tumor-node-metastasis (TNM) framework. Several diseases have incorporated nonanatomic prognostic factors into the determination of TNM staging groups.

**Objective:**

To refine TNM staging groups for Epstein-Barr virus (EBV)–related nonmetastatic nasopharyngeal carcinoma (NPC) by incorporating EBV DNA status.

**Design, Setting, and Participants:**

This multicenter prognostic study included patients with NPC treated with radiotherapy at 2 hospitals in China from January 2008 to December 2016. Progression-free survival and overall survival according to EBV DNA status and the TNM staging system were compared. Recursive partitioning analysis (RPA) combined with supervised clustering was applied to derive prognostic groupings, and then a refined RPA staging schema was developed, validated, and compared with existing staging schemes. Statistical analyses were conducted from October 1, 2020, to June 15, 2021.

**Exposures:**

Curative intensity-modulated radiotherapy with or without platinum-based chemotherapy.

**Main Outcomes and Measures:**

The primary end point was progression-free survival. The performance of the staging system was assessed using the time-dependent area under the receiver operating characteristic curves and the TNM stage system’s evaluation methodology.

**Results:**

A total of 2354 patients (1709 men [72.6%]; median [interquartile range] age, 45 [38-53] years) were split into training (1372 [58.3%]), internal validation (672 [28.5%]), and external validation (310 [13.2%]) cohorts. Pretreatment EBV DNA was detected in 1338 (56.8%) patients. EBV DNA status was an independent prognostic factor: lower survival probability by higher TNM stage was evident in EBV DNA–positive patients but not in those with EBV DNA–negative disease. After integrating EBV DNA status and TNM stage, nonmetastatic NPC cases were categorized into RPA-I (T1-3N0 or EBV DNA–negative T1-3N1 cancers), RPA-II (EBV DNA–positive T1-3N1-2 or EBV DNA–negative T1-3N2-3/T4N0-3 cancers), and RPA-III (EBV DNA–positive T4N0-3/T1-3N3 cancers) groups, each with distinctly different prognosis. This system of RPA staging outperformed the current TNM stage system and 2 reported RPA staging schemes. These results were internally and externally validated.

**Conclusions and Relevance:**

An RPA-based staging system for EBV-related NPC cases was associated with improved outcomes. This staging system may facilitate prognostic stratification and clinical trial designs.

## Key Points

**Question:**

Can incorporating Epstein-Barr virus DNA status into analysis of the tumor-node-metastasis framework improve cancer staging quality in nasopharyngeal carcinoma?

**Findings:**

In this multicenter prognostic study of 2354 patients in China, a recursive partitioning analysis (RPA)–based staging system that divided nonmetastatic nasopharyngeal carcinoma cases into 3 groups with distinctly different prognoses was developed and validated. The RPA stage system outperformed the current TNM staging and 2 reported RPA staging schemes.

**Meaning:**

These results suggest that an RPA-based staging system can outperform conventional TMN staging groups for predicting survival rates.

## Teaser

This prognostic study describes the development and validation of a novel staging system for nonmetastatic nasopharyngeal carcinoma based on recursive partitioning analysis of Epstein-Barr virus status.

## Introduction

For decades, the anatomic tumor-node-metastasis (TNM) staging system has been the most definitive solution for determining cancer’s anatomic extent and making treatment recommendations. The concept that nonanatomic prognostic factors complement the traditional anatomic staging system is widely accepted.^1,2,3,4,5^ The American Joint Committee on Cancer (AJCC)/Union for International Cancer Control (UICC) staging system in the *AJCC Cancer Staging Manual*, 8th edition (*AJCC* 8) emphasizes a personalized medicine approach.^6^ It features a trend toward expanding relevant molecular biomarkers to make accurate risk stratification. To date, only few cancers have included nonanatomic prognostic factors in their TNM stage groupings, such as prostate cancer, esophagus cancer, and gestational trophoblastic tumors.

Epstein-Barr virus (EBV) infection is the predominant etiological factor for nasopharyngeal carcinoma (NPC).^7^ Growing evidence reveals that circulating EBV DNA load reflects tumor burdens and biological properties.^8,9,10,11^ Elevated baseline circulating EBV DNA is associated with unfavorable clinical outcomes.^12^ This quantitative biomarker is useful in population screening, disease diagnosis, treatment monitoring, and posttherapy surveillance.^7,13,14,15,16^ However, the existing NPC cancer stage has not yet distinguished the 2 subtypes of EBV DNA–negative and EBV DNA–positive diseases. The revision of the current TNM staging system by incorporating EBV DNA status is needed.^7^ Several exploratory studies aimed at integrating EBV DNA level into the TNM framework have reported promising results.^17,18,19^

In this multicenter prognostic study, we first verified the prognostic value of EBV DNA. Next, we evaluated the *AJCC* 8 TNM staging system’s prediction performance for EBV DNA–negative and EBV DNA–positive NPC. Finally, we developed and validated a modified staging system based on current TNM classifications and EBV DNA status using recursive partitioning analysis (RPA).

## Methods

### Study Design and Patients

This multicenter, retrospective prognostic study was conducted at 2 large-scale hospitals in endemic regions in China. The primary cohort was derived from the Sun Yat-Sen University Cancer Center and comprised 2044 patients with newly diagnosed NPC treated between January 2008 and December 2016. We randomly assigned the primary data set into a training cohort (1372 patients) and an internal validation cohort (672 patients). A separate cohort from the First People's Hospital of Foshan (310 patients), which included consecutive patients treated between January 2010 and December 2011, was set as the external validation data set. Details of inclusion and exclusion criteria of the study are provided in the eAppendix in the Supplement. The study was performed under the Declaration of Helsinki and followed the Transparent Reporting of a Multivariable Prediction Model for Individual Prognosis or Diagnosis (TRIPOD) reporting guideline. The Chinese Ethics Committee of Registering Clinical Trials approved this study (ChiECRCT20200461). Informed consent was waived because of the study’s retrospective nature and the anonymization of individual data.

### Treatment Protocol

Patients were treated based on the *AJCC *6 or *AJCC *7 edition of the AJCC staging system, which represented the staging manual used for medical care. We centrally restaged patients based on the *AJCC *8.^6^ Plasma EBV DNA titer was measured using a real-time quantitative polymerase chain reaction assay. More details regarding the test method have been described in eAppendix in the Supplement. According to the institutional treatment protocol, radiotherapy alone was recommended for early-stage disease. Concomitant chemoradiotherapy combined or not combined with induction/adjuvant chemotherapy was recommended for locoregionally advanced disease. All patients received definitive radiotherapy using the intensity-modulated radiation therapy (IMRT) technique. Target volumes were delineated regarding our institutional treatment guidelines and the International Commission on Radiation Units and Measurements Reports 50 and 62.^20,21^ The prescribed doses were 66 to 72 Gray equivalent (GyE) in 30 to 33 fractions to the primary gross tumor volume and 64 to 70 GyE in 30 to 33 fractions to the involved nodal regions as previously described.^22^

### Study End Points

Our primary end point was progression-free survival (PFS), calculated from the date of diagnosis to the date of disease progression or censorship at the last follow-up. Disease progression was defined as the presence of a newly detected local or regional relapse or distant metastasis confirmed by biopsy or radiologic images and death from any cause. Our secondary end point was overall survival (OS), measured from the date of diagnosis to the date of death from any causes or censorship at the last follow-up. Patients lost to follow-up are censored at the date of last contact.

### Statistical Analysis

Survival probabilities were estimated using the Kaplan-Meier approach, and survival differences were compared using a log-rank test. Hazard ratios (HRs) and the corresponding 95% CIs were calculated using Cox proportional hazard models. We applied the inverse probability weighting (IPW) method to reduce the impact of confounders (eAppendix in the Supplement). The new staging system's construction and evaluation follow the AJCC Personalized Medicine Core checklist.^23^ First, RPA was performed in the EBV DNA–negative and EBV DNA–positive patients in the training cohort separately. We obtained several clusters with different PFS probabilities based on survival decision-making trees constructed in the autoRPA server.^24^ Next, we measured the similarity between each cluster pair using Spearman correlation distance based on relative intergroup HRs and pairwise log-rank *P* values (corresponding to the PFS). We constructed an RPA-based staging by reducing clusters with the supervised clustering method to add clinical convenience. Then, we estimated the new staging system’s predictive accuracy using the time-dependent area under the receiver operating characteristic curves (tAUC) and assessed calibration using calibration plots. Finally, our refined RPA stage system’s cancer evaluative quality was compared with the *AJCC *8 TNM system and 2 reported RPA stages using 5 widely accepted evaluation methodology refined by Xu and colleagues.^17,18,25^ Evaluation criteria consisted of hazard consistency, hazard discrimination, explained variation, likelihood difference, and balance. We ranked the stage schemas using summarized scores, with the lowest score ranking first. The results were validated by using 1000 bootstrap replicates and 2 independent validation data sets. All statistical analyses were conducted using the R version 4.0.5 (R Project for Statistical Computing). A 2-sided *P* < .05 was considered to be significant.

## Results

### Patient Characteristics

This study included 2354 patients (1709 men [72.6%]; median [interquartile range] age, 45 [38-53] years) split into training (1372 [58.3%]), internal validation (672 [28.5%]), and external validation (310 [13.2%]) cohorts. Patient characteristics of the study population are summarized in Table 1. The median follow-up time was 56.8 months (range, 2.7–128 months). All patients underwent definitive radiotherapy using the IMRT technique, and platinum-containing chemotherapy was administered to 2072 (88.0%) patients. Plasma EBV DNA was detected in 1338 (56.8%) patients (eTable in the Supplement). EBV DNA levels significantly correlated with the patient’s TNM classifications. The median (interquartile range) EBV DNA titer for each stage were: stage I, 0 (0-0); stage II, 0 (0-350); stage III, 100 (0-4520); and stage IVa, 2130 (0-17875).

**Table 1.** Patient Characteristics in the Training and Validation Data Sets

Characteristic	Patients, No. (%)		
	Training set (N = 1372)	Internal validation set (N = 672)	External validation set (N = 310)
Age, median (IQR), y	45.0 (38.0-52.0)	45.0 (37.0-54.0)	47.0 (40.0-56.0)
Sex			
Women	374 (27.3)	179 (26.6)	92 (29.7)
Men	998 (72.7)	493 (73.4)	218 (70.3)
BMI, median (IQR)	20.9 (19.1-23.2)	20.6 (19.0-23.3)	20.4 (17.9-22.9)
Smoking			
No	880 (64.1)	447 (66.5)	273 (88.1)
Yes	492 (35.9)	225 (33.5)	37 (11.9)
Histology			
Type II	30 (2.2)	18 (2.7)	8 (2.6)
Type III	1342 (97.8)	654 (97.3)	302 (97.4)
Tumor category			
T1	104 (7.6)	34 (5.1)	40 (12.9)
T2	184 (13.4)	94 (14.0)	80 (25.8)
T3	732 (53.4)	363 (54.0)	98 (31.6)
T4	352 (25.7)	181 (26.9)	92 (29.7)
Node category			
N0	174 (12.7)	92 (13.7)	48 (15.5)
N1	544 (39.7)	244 (36.3)	87 (28.1)
N2	472 (34.4)	232 (34.5)	128 (41.3)
N3	182 (13.3)	104 (15.5)	47 (15.2)
Clinical stage			
I	37 (2.70)	13 (1.9)	11 (3.6)
II	132 (9.6)	60 (8.9)	44 (14.2)
III	718 (52.3)	338 (50.3)	129 (41.6)
IVa	485 (35.3)	261 (38.8)	126 (40.6)
Treatment			
IMRT alone	177 (12.9)	60 (8.9)	45 (14.5)
CRT	1195 (87.1)	612 (91.1)	265 (85.5)
EBV DNA status			
Negative	557 (40.6)	265 (39.4)	194 (62.6)
Positive	815 (59.4)	407 (60.6)	116 (37.4)
Censored patients	1117 (81.4)	551 (82.0)	223 (71.9)
Progression events	255 (18.6)	121 (18.0)	87 (28.1)
Death events	130 (9.5)	71 (10.6)	68 (21.9)

Abbreviations: CRT, chemoradiotherapy; BMI, body mass index (calculated as weight in kilograms divided by height in meters squared); EBV, Epstein-Barr virus; IMRT, intensity-modulated radiation therapy; IQR, interquartile range.

### Prognostic Value of EBV DNA Status

Unadjusted Kaplan-Meier survival analyses conducted in the entire cohort showed that, compared with patients with EBV DNA–negative NPC, patients with EBV DNA–positive NPC had worse PFS (5-year PFS: 72.6% [95% CI, 70.0-75.3] vs 89.6% [95% CI, 87.8-91.8]; *P* < .001) and OS (5-year OS: 84.7% [95% CI, 82.5-86.9] vs 94.6% [95% CI, 92.9-96.2]; *P* < .001) (eFigure 1 in the Supplement). After adjusting for confounders using the IPW method, sufficient covariate balances were achieved between patients with EBV DNA–negative and EBV DNA–positive NPC, with all standardized mean differences reducing to less than 0.1 (eFigure 2 in the Supplement). IPW-adjusted Kaplan-Meier survival analyses revealed that EBV DNA status was an unfavorable prognostic factor for PFS (5-year PFS: EBV DNA–positive NPC, 75.4% [95% CI, 72.9-78.0] vs EBV DNA–negative, 87.7% [95% CI, 85.2-90.2]; *P* < .001) and OS (5-year OS: EBV DNA–positive NPC, 86.3% [95% CI, 84.2-88.3] vs EBV DNA–negative, 93.3% [95% CI, 91.2-95.4]; *P* < .001) (eFigure 1 in the Supplement). The prognostic value of EBV DNA status for patients with stage I to IVa NPC was further examined in subgroup analyses and visualized in a forest plot (eFigure 3 in the Supplement).

### Performance of the *AJCC *8 TNM Stage Scheme in EBV DNA–Negative and EBV DNA–Positive Disease

The current TNM stage was inadequate for patients with EBV DNA–negative NPC to provide a prognosis, although it was acceptable for patients with EBV DNA–positive NPC. The survival disparities between the T and N categories were not distinct for patients with EBV DNA–negative NPC, but were apparent for those with EBV DNA–positive diseases (eFigure 4 and eFigure 5 in the Supplement). There was no significant difference in 5-year PFS in EBV DNA–negative NPC patients with stage I, II, and III diseases, although the difference was still significantly lower for those with stage IVa disease (80.4%; 95% CI, 75.0-86.0; *P* = .002) (eFigure 6 in the Supplement). There were no distinct differences in 5-year OS in EBV DNA–negative NPC patients with stage I, II, III, and IVa disease (eFigure 6 in the Supplement). On the contrary, EBV DNA–positive NPC patients had a relatively monotonic reduction in PFS and OS with increasing TNM classifications. The 5-year PFS probability of patients with TNM stage of I, II, III, and IVa was 100% (95% CI, 100%-100%), 93.0% (95% CI, 87.1%-99.3%), 77.1% (95% CI, 73.5%-80.9%), and 64.5% (95% CI, 60.5%-68.9%), respectively (*P* < .001) (eFigure 6 in the Supplement). The 5-year OS probability of patients with TNM stage of I, II, III, and IVa was 100% (95% CI, 100%-100%), 96.8% (95% CI, 92.3%-100%), 86.9% (95% CI, 84.0%-90.0%), and 80.2% (95% CI, 76.7%-83.9%), respectively (*P* < .001).

### Refined TNM Stage Groupings Based on EBV DNA Status

**Figure 1.**
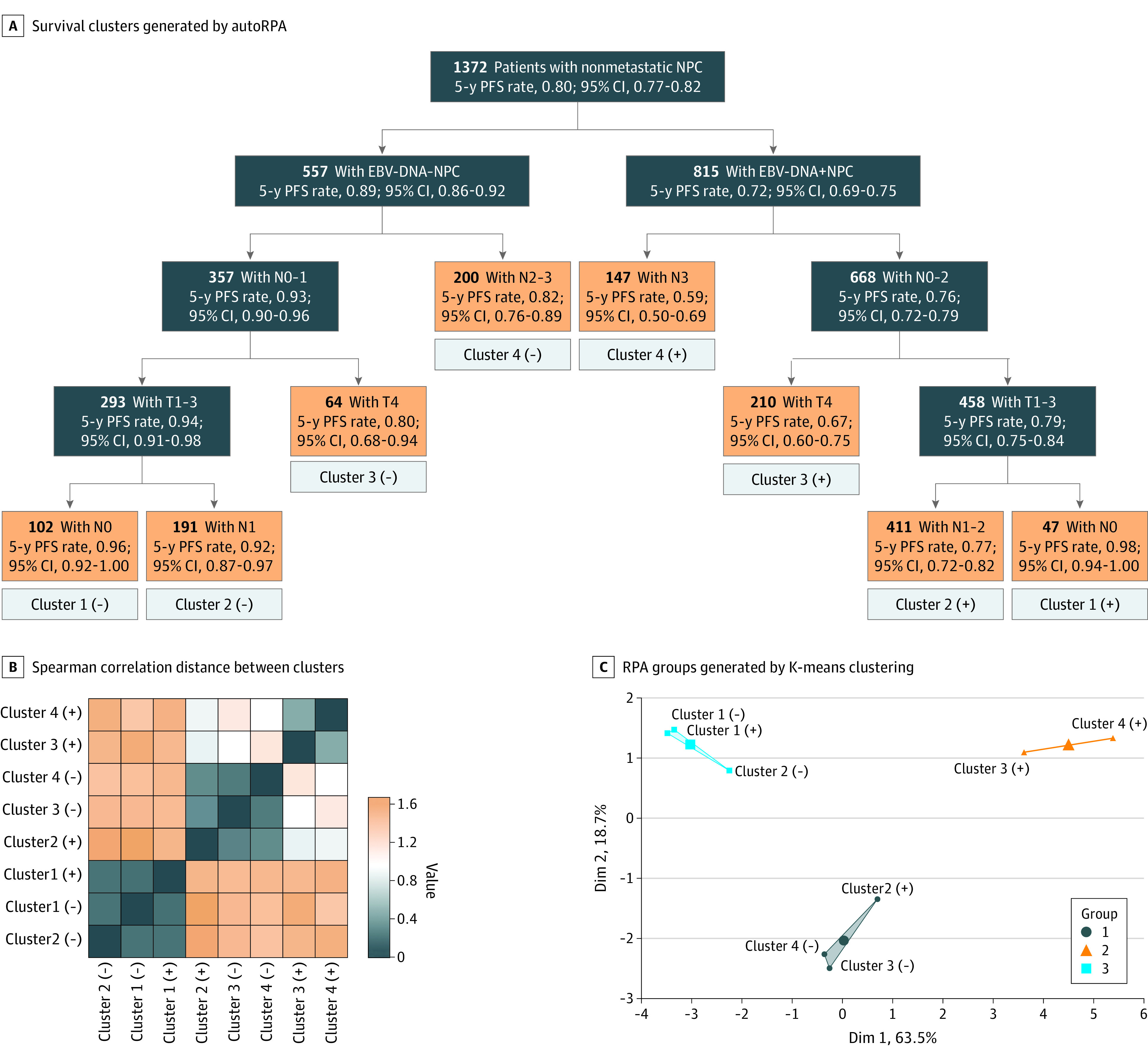
Development of the Survival Clusters and Generation of the RPA Groupings In panel C, the factoextra package in R software was used to visualize k-means clusters. Observations were represented by points in the resulting plot, using principal components if the number of variables is greater than 2.

**Figure 2.**
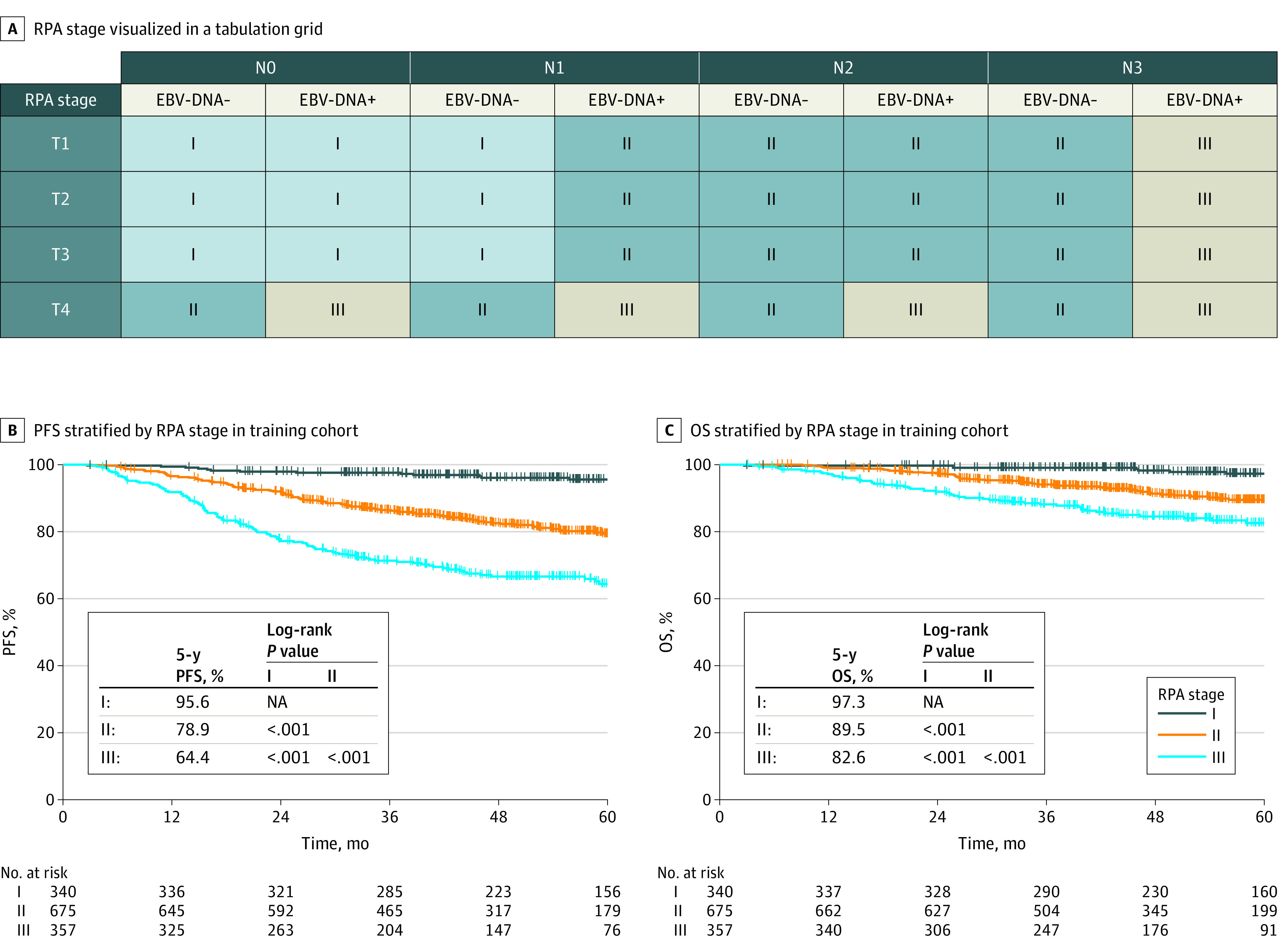
Visualization of the RPA Stage and Kaplan-Meier Survival Analyses Stratified by RPA Stage in the Training Cohort EBV indicates Epstein-Barr virus; OS, overall survival; PFS, progression-free survival; RPA, recursive partitioning analysis.

In the Sun Yat-Sen training set, the autoRPA web server generated survival trees containing 8 survival clusters in EBV DNA–negative and EBV DNA–positive population. The 5-year PFS probabilities by these clusters in EBV DNA–negative and EBV DNA–positive patients are shown in Figure 1. We measured the distance or the similarity between each pair of clusters using Spearman correlation distance based on relative intergroup HRs and pairwise log-rank *P* values. We further reduced the number of survival clusters using the supervised clustering method based on the distance. The optimal number of clusters determined by the silhouette method was 3 (eFigure 7 in the Supplement). Two supervised clustering methods generated identical clusters based on the optimal number of clusters, including k-means clustering (Figure 1) and hierarchical clustering (eFigure 8 in the Supplement). Therefore, we derived a refined stage grouping based on the above clusters (Figure 2). The proposed RPA stage system divided nonmetastatic NPC into RPA-I (low risk: T1-3N0 or EBV DNA–negative T1-3N1), RPA-II (moderate risk: EBV DNA–positive T1-3N1-2 or EBV DNA–negative T1-3N2-3/T4N0-3), and RPA-III (high risk: EBV DNA–positive T4N0-3/T1-3N3).

### Performance Comparison of Different Cancer Staging Systems

The RPA staging scheme showed a monotonic reduction concerning PFS and OS when the disease stage was increased. There were significant differences across patients with RPA stage I, II, and III diseases in the training cohort (all pairwise *P* < .05, Figure 2). The 5-year PFS and OS probabilities of RPA stage I, II, and III were 95.6% (95% CI, 93.2%-98.0%) and 97.3% (95% CI, 95.3%-99.3%), 78.9% (95% CI, 75.3%-82.6%) and 89.5% (95% CI, 86.8%-92.3%), and 64.4% (95% CI, 59.0%-70.2%) and 82.6% (95% CI, 78.2%-87.3%). We observed similar findings in the internal validation set and Foshan external validation set (eFigure 9 in the Supplement). The RPA staging system reclassified patients with stage II to IVa of the existing TNM staging system except for stage I (eFigure 10 in the Supplement). The addition of EBV DNA status into the TNM classifications significantly improved the survival prediction performance of the RPA system. The RPA system had the highest tAUCs for PFS and OS prediction in the SYSUCC training cohort, which was significantly higher than the *AJCC* 8 TNM stage (mean [SD] tAUC at 5-year for PFS: 0.715 [0.019] vs 0.674 [0.019], *P* < .001; tAUC at 5-year for OS: 0.687 [0.025] vs 0.641 [0.025], *P* = .003) (Table 2). The RPA stage was well calibrated for predicting PFS at various time points in the training set (eFigure 11 in the Supplement). Similar findings were observed in 2 validation cohorts (Table 2; eFigure 11 in the Supplement). The proposed RPA staging outperformed the TNM staging system and the 2 previously reported RPA staging groups. The 2 independent validation data sets and the internal bootstrap validation also provided similar results (Table 3).

**Table 2.** Comparison of Prediction Performance for Survival in Different Staging Models^a^

Staging Models	SYSUCC training set				SYSUCC internal validation set				Foshan external validation set			
	tAUC at 3y, mean (SD)	*P* value	tAUC at 5y, mean (SD)	*P* value	tAUC at 3y, mean (SD)	*P* value	tAUC at 5y, mean (SD)	*P* value	tAUC at 3y, mean (SD)	*P* value	tAUC at 5y, mean (SD)	*P* value
PFS												
RPA stage	0.705 (0.017)	[Reference]	0.715 (0.019)	[Reference]	0.679 (0.024)	[Reference]	0.691 (0.027)	[Reference]	0.690 (0.034)	[Reference]	0.690 (0.033)	[Reference]
TNM stage	0.660 (0.018)	<.001	0.674 (0.019)	<.001	0.647 (0.025)	.04	0.644 (0.028)	.007	0.663 (0.036)	.37	0.679 (0.034)	.70
RPA (Guo)^18^ stage	0.674 (0.019)	.02	0.688 (0.020)	.07	0.676 (0.026)	.86	0.683 (0.028)	.74	0.655 (0.041)	.29	0.671 (0.036)	.50
RPA (Lee)^17^ stage	0.652 (0.020)	.001	0.063 (0.020)	.62	0.629 (0.028)	.04	0.669 (0.028)	.44	0.627 (0.044)^b^	.06^b^	0.623 (0.038)^b^	.02^b^
OS												
RPA stage	0.685 (0.025)	[Reference]	0.687 (0.025)	[Reference]	0.710 (0.029)	[Reference]	0.691 (0.034)	[Reference]	0.676 (0.063)	[Reference]	0.686 (0.049)	[Reference]
TNM stage	0.641 (0.026)	.001	0.641 (0.025)	.003	0.661 (0.033)	.004	0.648 (0.033)	.03	0.560 (0.063)	<.001	0.625 (0.049)	.047
RPA (Guo)^18^ stage	0.673 (0.019)	.72	0.686 (0.020)	.97	0.672 (0.034)	.27	0.629 (0.036)	.04	0.570 (0.067)	.01	0.623 (0.050)	.047
RPA (Lee)^17^ stage	0.650 (0.020)	.28	0.664 (0.021)	.48	0.623 (0.037)	.02	0.636 (0.036)	.10	0.648 (0.061)^b^	.54^b^	0.656 (0.047)^b^	.42^b^

Abbreviations: OS, overall survival; PFS, progression-free survival; RPA, recursive partitioning analysis; SYSUCC, Sun Yat-Sen University Cancer Center; tAUC, time-independent area under the receiver operating characteristic curves; TNM, tumor-node-metastasis staging according to the American Joint Committee on Cancer’s *Cancer Staging Manual*, 8th edition.^6^

^a^ For each time point, the compare function in the timeROC package provided by the R software computes the difference between estimated AUCs of both markers, the variance of the difference using the independent and identically distributed representation of the AUC estimators, and returns the *P* value of the comparison test.

^b^ According to the laboratory practice standards used in the First Hospital of Foshan, an EBV DNA titer less than 1000 was reported as negative and not given an actual value. As a compromise, we adopted the nearest integer (1000) as a substitute in the calculation procedure.

**Table 3.** Comparison of Performance of the Different RPA Staging Systems and TNM Staging System Under 5 Evaluation Criteria

Criteria	SYSUCC training set				SYSUCC internal validation set				Foshan external validation set				Bootstrap validation set			
	RPA staging	RPA staging 1^18^	RPA staging 2^17^	TNM staging	RPA staging	RPA staging 1^18^	RPA staging 2^17^	TNM staging	RPA staging	RPA staging 1^18^	RPA staging 2^17^	TNM staging	RPA staging	RPA staging 1^18^	RPA staging 2^17^	TNM staging
**Hazard consistency** (similarity of survival rate for subgroups within each stage group)																
Standardized score	0.00	0.23	1.00	0.85	0.00	0.12	0.66	1.00	0.00	0.52	1.00^a^	0.67	0.00	0.25	1.00	0.75
Rank	1	2	4	3	1	2	3	4	1	2	4^a^	3	1	2	4	3
**Hazard discrimination** (differences in survival rate across stage groups)																
Standardized score	1.00	0.44	0.92	0.00	0.92	0.14	1.00	0.00	1.00	0.00	0.28^a^	0.08	1.00	0.28	0.64	0.00
Rank	4	2	3	1	3	2	4	1	4	1	3^a^	2	4	2	3	1
**Likelihood difference** (difference in goodness-of-fit between models)																
Standardized score	0.00	0.83	1.00	0.93	0.00	0.81	0.84	1.00	0.00	0.92	1.00^a^	0.83	0.00	0.80	1.00	0.88
Rank	1	2	4	3	1	2	3	4	1	3	4^a^	2	1	2	4	3
**Explained variance** (percentage of variation in survival rate accounted for by stage groupings)																
Standardized score	0.00	0.41	1.00	0.80	0.00	0.14	0.89	1.00	0.00	0.55	1.00^a^	0.60	0.00	0.38	1.00	0.74
Rank	1	2	4	3	1	2	3	4	1	2	4^a^	3	1	2	4	3
**Balance** (difference in sample sizes across stage groups)																
Standardized score	0.00	0.37	0.96	1.00	0.00	0.32	0.81	1.00	0.09	0.00	1.00^a^	0.81	0.00	0.37	0.94	1.00
Rank	2	1	3	4	1	2	3	4	2	1	4^a^	3	2	1	3	4
**Overall**																
Score	1.00	2.29	4.87	3.58	0.92	1.53	4.20	4.00	1.09	1.99	4.28^a^	2.99	0.71	2.15	4.52	3.54
Rank	1	2	4	3	1	2	4	3	1	2	4^a^	3	1	2	4	3

Abbreviations: RPA, recursive partitioning analysis; SYSUCC, Sun Yat-Sen University Cancer Center; TNM, tumor-node-metastasis.

^a^ According to the laboratory practice standards used in the First Hospital of Foshan, an EBV DNA titer less than 1000 was reported as negative and not given an actual value. As a compromise, we adopted the nearest integer (1000) as a substitute in the calculation procedure.

We found that patients with EBV DNA–negative NPC at advanced stages (ie, stages III and IVa) did not experience the same poor outcomes as those with EBV DNA–positive disease. Fewer than 10% of patients with stage III EBV DNA–negative NPC would experience tumor progression at 5 years compared with more than 20% with EBV DNA–positive disease. A similar finding was observed in patients with stage IVa disease. In our study, N0 and EBV DNA negativity seemed to be favorable characteristics. Patients with N0 or EBV DNA–negative NPC were downstaged in our proposed RPA staging system. For example, 172 patients (72.9%) with stage II NPC (T2N0 or EBV DNA–negative T1-2N1) in the current TNM staging system were relegated to RPA stage I, whereas 342 patients (28.9%) with stage III NPC (T3N0 or EBV DNA–negative T3N1) were relegated to RPA stage I. On the contrary, T4 is an adverse factor regardless of the N category, as is N3 irrespective of the T category. They both represented the highest risk group in nonmetastatic NPC (M0 disease). Nevertheless, the protective effect of EBV DNA–negative was still evident in these high-risk patients. EBV DNA–negative T4 or N3 were relegated to RPA stage II, whereas EBV DNA–positive T4 or N3 were classified as RPA stage III.

## Discussion

The AJCC and the UICC have been striving to introduce nonanatomic factors into the TNM framework to differentiate prognosis further.^26^ In the past decades, numerous studies have provided compelling evidence that circulating EBV DNA is closely related to the tumor burden and could be a robust prognostic biomarker in NPC.^8,9,10,11,27,28^ EBV DNA is significant in survival prediction and risk-stratified treatment adaptation in the clinic.^13^ Our results agreed with these studies. Patients with EBV DNA–positive NPC had worse survival outcomes than those with EBV DNA–negative NPC. EBV DNA status was shown to be a significant independent prognostic factor, even after adequate adjustment was made for confounding factors. Undoubtedly, this survival disparity hampers medical outcome studies and clinical trial design and affects treatment recommendations.

Human papillomavirus (HPV)-positive oropharyngeal cancer (OPC) is a typical virus-related cancer, which differs from tobacco-related and alcohol-related (ie, HPV-negative) OPC. Patients with HPV-positive OPC would not experience the same poor prognosis as those with HPV-negative disease. The AJCC/UICC TNM staging system is acceptable for HPV-negative OPC to depict prognosis but does not perform well in HPV-positive OPC. Two previous studies have introduced RPA-based TNM stage groupings for HPV-positive OPC, allowing for a more accurate depiction of survival outcomes.^4,29^ Like OPC, the staging scheme should be more flexible for virus-related NPC, as EBV DNA–negative and EBV DNA–positive NPC are biologically different diseases with significantly different prognoses and potentially require different treatment strategies. However, the *AJCC* 8 TNM stage for NPC does not distinguish between these 2 subtypes of diseases. Indeed, the current TNM staging system did not reflect NPCs prognosis well in our study, especially for those with EBV DNA–negative NPC. Given the robust evidence supporting the prognostic significance of plasma EBV DNA, the TNM staging group’s modifications adapted to incorporate this molecular biomarker are warranted.

Our study population comprised patients with unselected nonmetastatic NPC treated with curative IMRT, representing the present clinical practice benchmark. The proposed RPA staging system outperformed the existing AJCC/UICC staging system in survival prediction and staging performance evaluation under the current treatment paradigm. Our result was similar to 2 previous studies investigating RPA-based TNM stage schemes for NPC to some extent.^17,18^ Incorporating EBV DNA into the TNM staging system improved the predictive ability and stage quality. However, the essential difference between our study and the 2 previous studies was that we regarded different EBV DNA NPC results as 2 subtypes of diseases. We hypothesized HRs for the ordinal T and N categories within EBV DNA–negative and EBV DNA–positive NPC could be inconsistent. To make the current TNM staging system more appropriate and clinically appliable, we separately determined TNM stage groupings for EBV DNA–negative and EBV DNA–positive NPC in developing the proposed RPA stage system. In comparison, the 2 previous studies regarded EBV DNA as an additional grouping factor, similar to the T or N category, in generating their RPA staging schemes. Apart from the methodological differences, we deem that the difference between negative and positive status is more discriminative than EBV DNA levels in measuring NPC severity. Because high or low levels of EBV DNA were usually determined by a statistical cutoff and were highly varied because heterogeneity in study population and detection technique, the adoption of EBV DNA status in our study might reduce the influence of highly varied cutoffs of EBV DNA because of interlaboratory differences in detection and heterogeneous study population. Encouragingly, our RPA stage with the 3 risk groupings outperformed the 2 reported RPA schemes with 4- and 5-tier risk stratifications. The improved performances were validated across different data sets.

### Limitations

This study had several limitations. First, the study population was purely derived from endemic regions. Geographically, the distribution of NPC subtypes differs significantly throughout the world. Keratinizing NPC is more frequently reported in nonendemic areas, whereas the nonkeratinizing subtype predominates in endemic regions.^7,30^ Therefore, the new staging system’s generalization into patients from the nonendemic areas needs further validation. Second, previous studies have also reported that even for the same quantitative assay using identical procedures, the interlaboratory measurement of EBV DNA could be heterogeneous due to nonstandardized and nonharmonized techniques.^13,31^ However, the standardization and harmonization of EBV DNA measurements among different institutions are extremely difficult to accomplish. The staging strategy we adopted might reduce these influences to some extent. Indeed, we have shown concordant results across participating institutions in heterogeneity assessment.

## Conclusions

We developed and validated an RPA-based staging system for EBV-related nonmetastatic NPC based on EBV DNA status. Our RPA staging system outperformed the *AJCC* 8 TNM staging system and 2 published RPA staging schemes. Our new staging system may optimize prognostic stratification and facilitate clinical trial designs and survival prediction for future studies. Independent external validation of the proposed RPA stage among patients from nonendemic areas is warranted.

## Notes

Supplement. **eAppendix.** Supplementary Methods **eTable.** Characteristics of the 2354 Patients with Nonmetastatic Nasopharyngeal Carcinoma Stratified by EBV DNA Status in the Entire Cohort **eFigure 1.** Unadjusted Kaplan-Meier Survival Analyses of Progression-Free Survival and Overall Survival Stratified by the EBV DNA Status; Inverse Probability Weighting (IPW)-Adjusted Kaplan-Meier Survival Analyses of Progression-Free Survival and Overall Survival Stratified by the EBV DNA Status **eFigure 2.** Covariate Balances Between Patients With EBV DNA (-) and EBV DNA (+) NPC Assessed Using Standardized Mean Differences (SMDs) **eFigure 3.** Prognostic Value of EBV DNA Status for Patients With Stage I to IVa in Subgroup Analyses **eFigure 4.** Kaplan-Meier Survival Analyses for Progression-Free Survival and Overall Survival Stratified by the T Category in Patients With EBV DNA (-) NPC and Patients With EBV DNA (+) NPC **eFigure 5.** Kaplan-Meier Survival Analyses for Progression-Free Survival and Overall Survival Stratified by the N Category in Patients With EBV DNA (-) NPC and Patients With EBV DNA (+) NPC **eFigure 6.** Kaplan-Meier Survival Analyses for Progression-Free Survival and Overall Survival Stratified by the 8th Edition of the TNM Staging System in Patients With EBV DNA (-) NPC and Patients With EBV DNA (+) NPC **eFigure 7.** Optimal Number of Clusters Determined by the Silhouette Method **eFigure 8.** RPA groups Generated by Hierarchical Clustering **eFigure 9.** Kaplan-Meier Survival Analyses Stratified by the RPA Stage in the Validation Cohorts **eFigure 10.** Reclassification of the 8th TNM Stage Using the Proposed RPA staging system **eFigure 11.** Calibration Plots for Predicting 3- and 5-Year PFS in the Sun Yat-Sen University Cancer Center training cohort, Sun Yat-Sen University Cancer Center Internal Validation Cohort, and Foshan External Validation Cohort

## References

[1] Patel SG, Shah JP. TNM staging of cancers of the head and neck: striving for uniformity among diversity. CA Cancer J Clin. 2005;55(4):242-258. doi:10.3322/canjclin.55.4.242 16020425

[2] Takes RP, Rinaldo A, Silver CE, . Future of the TNM classification and staging system in head and neck cancer. Head Neck. 2010;32(12):1693-1711. doi:10.1002/hed.21361 20191627

[3] Amin MB, Greene FL, Edge SB, . The Eighth Edition AJCC Cancer Staging Manual: continuing to build a bridge from a population-based to a more “personalized” approach to cancer staging. CA Cancer J Clin. 2017;67(2):93-99. doi:10.3322/caac.21388 28094848

[4] O’Sullivan B, Huang SH, Su J, . Development and validation of a staging system for HPV-related oropharyngeal cancer by the International Collaboration on Oropharyngeal cancer Network for Staging (ICON-S): a multicentre cohort study. Lancet Oncol. 2016;17(4):440-451. doi:10.1016/S1470-2045(15)00560-4 26936027

[5] Dess RT, Suresh K, Zelefsky MJ, . Development and validation of a clinical prognostic stage group system for nonmetastatic prostate cancer using disease-specific mortality results from the International Staging Collaboration for Cancer of the Prostate. JAMA Oncol. 2020;6(12):1912-1920. doi:10.1001/jamaoncol.2020.4922 33090219 PMC7582232

[6] Amin MB, Edge S, Greene F, , eds. AJCC Cancer Staging Manual, 8th edition. Springer; 2017.

[7] Chen Y-P, Chan ATC, Le Q-T, Blanchard P, Sun Y, Ma J. Nasopharyngeal carcinoma. Lancet. 2019;394(10192):64-80. doi:10.1016/S0140-6736(19)30956-0 31178151

[8] Ma BB, King A, Lo YM, . Relationship between pretreatment level of plasma Epstein-Barr virus DNA, tumor burden, and metabolic activity in advanced nasopharyngeal carcinoma. Int J Radiat Oncol Biol Phys. 2006;66(3):714-720. doi:10.1016/j.ijrobp.2006.05.064 17011447

[9] Leung SF, Zee B, Ma BB, . Plasma Epstein-Barr viral deoxyribonucleic acid quantitation complements tumor-node-metastasis staging prognostication in nasopharyngeal carcinoma. J Clin Oncol. 2006;24(34):5414-5418. doi:10.1200/JCO.2006.07.7982 17135642

[10] Lin JC, Wang WY, Chen KY, . Quantification of plasma Epstein-Barr virus DNA in patients with advanced nasopharyngeal carcinoma. N Engl J Med. 2004;350(24):2461-2470. doi:10.1056/NEJMoa032260 15190138

[11] Tang LQ, Li CF, Li J, . Establishment and validation of prognostic nomograms for endemic nasopharyngeal carcinoma. J Natl Cancer Inst. 2015;108(1):djv291. doi:10.1093/jnci/djv291 26467665

[12] Zhang W, Chen Y, Chen L, . The clinical utility of plasma Epstein-Barr virus DNA assays in nasopharyngeal carcinoma: the dawn of a new era?: a systematic review and meta-analysis of 7836 cases. Medicine (Baltimore). 2015;94(20):e845. doi:10.1097/MD.0000000000000845 25997061 PMC4602858

[13] Kim KY, Le QT, Yom SS, . Clinical utility of Epstein-Barr Virus DNA testing in the treatment of nasopharyngeal carcinoma patients. Int J Radiat Oncol Biol Phys. 2017;98(5):996-1001. doi:10.1016/j.ijrobp.2017.03.018 28721913 PMC13360285

[14] Tsao SW, Tsang CM, Lo KW. Epstein-Barr virus infection and nasopharyngeal carcinoma. Philos Trans R Soc Lond B Biol Sci. 2017;372(1732). doi:10.1098/rstb.2016.0270 PMC5597737 28893937

[15] Li WZ, Lv SH, Liu GY, . Development of a prognostic model to identify the suitable definitive radiation therapy candidates in de novo metastatic nasopharyngeal carcinoma: a real-world study. Int J Radiat Oncol Biol Phys. 2021;109(1):120-130. doi:10.1016/j.ijrobp.2020.08.045 32853711 PMC9107935

[16] Li WZ, Hua X, Xie DH, . Prognostic model for risk stratification of de novo metastatic nasopharyngeal carcinoma patients treated with chemotherapy followed by locoregional radiotherapy. ESMO Open. 2021;6(1):100004. doi:10.1016/j.esmoop.2020.100004 33399071 PMC7807936

[17] Lee VH, Kwong DL, Choi CW, . The addition of pretreatment plasma Epstein-Barr virus DNA into the eighth edition of nasopharyngeal cancer TNM stage classification. Int J Cancer. 2019;144(7):1713-1722. doi:10.1002/ijc.31856 30192385

[18] Guo R, Tang LL, Mao YP, . Proposed modifications and incorporation of plasma Epstein-Barr virus DNA improve the TNM staging system for Epstein-Barr virus-related nasopharyngeal carcinoma. Cancer. 2019;125(1):79-89. doi:10.1002/cncr.31741 30351466

[19] Hui EP, Li WF, Ma BB, . Integrating postradiotherapy plasma Epstein-Barr virus DNA and TNM stage for risk stratification of nasopharyngeal carcinoma to adjuvant therapy. Ann Oncol. 2020;31(6):769-779. doi:10.1016/j.annonc.2020.03.289 32217076

[20] Chavaudra J, Bridier A. Definition of volumes in external radiotherapy: ICRU reports 50 and 62. Article in French. Cancer Radiother. 2001;5(5):472-478. doi:10.1016/s1278-3218(01)00117-2 11715299

[21] Li WF, Sun Y, Chen M, . Locoregional extension patterns of nasopharyngeal carcinoma and suggestions for clinical target volume delineation. Chin J Cancer. 2012;31(12):579-587. doi:10.5732/cjc.012.10095 22854064 PMC3777458

[22] Li WZ, Liu GY, Lin LF, . MRI-detected residual retropharyngeal lymph node after intensity-modulated radiotherapy in nasopharyngeal carcinoma: Prognostic value and a nomogram for the pretherapy prediction of it. Radiother Oncol. 2020;145:101-108. doi:10.1016/j.radonc.2019.12.018 31931288

[23] Kattan MW, Hess KR, Amin MB, ; members of the AJCC Precision Medicine Core. American Joint Committee on Cancer acceptance criteria for inclusion of risk models for individualized prognosis in the practice of precision medicine. CA Cancer J Clin. 2016;66(5):370-374. doi:10.3322/caac.21339 26784705 PMC4955656

[24] Xie Y, Luo X, Li H, . autoRPA: a web server for constructing cancer staging models by recursive partitioning analysis. Comput Struct Biotechnol J. 2020;18:3361-3367. doi:10.1016/j.csbj.2020.10.038 33294132 PMC7688999

[25] Xu W, Shen X, Su J, O’Sullivan B, Huang S. Refining evaluation methodology on TNM stage system: assessment on HPV-related oropharyngeal cancer. Austin Biom Biostat. 2015;2:1014.

[26] Kattan MW, Hess KR, Amin MB, ; members of the AJCC Precision Medicine Core. American Joint Committee on Cancer acceptance criteria for inclusion of risk models for individualized prognosis in the practice of precision medicine. CA Cancer J Clin. 2016;66(5):370-374. doi:10.3322/caac.21339 26784705 PMC4955656

[27] Wang WY, Twu CW, Chen HH, . Long-term survival analysis of nasopharyngeal carcinoma by plasma Epstein-Barr virus DNA levels. Cancer. 2013;119(5):963-970. doi:10.1002/cncr.27853 23065693

[28] Leung SF, Chan AT, Zee B, . Pretherapy quantitative measurement of circulating Epstein-Barr virus DNA is predictive of posttherapy distant failure in patients with early-stage nasopharyngeal carcinoma of undifferentiated type. Cancer. 2003;98(2):288-291. doi:10.1002/cncr.11496 12872347

[29] Huang SH, Xu W, Waldron J, . Refining American Joint Committee on Cancer/Union for International Cancer Control TNM stage and prognostic groups for human papillomavirus-related oropharyngeal carcinomas. J Clin Oncol. 2015;33(8):836-845. doi:10.1200/JCO.2014.58.6412 25667292

[30] Argirion I, Zarins KR, Ruterbusch JJ, . Increasing incidence of Epstein-Barr virus-related nasopharyngeal carcinoma in the United States. Cancer. 2020;126(1):121-130. doi:10.1002/cncr.32517 31524955 PMC6906241

[31] Le QT, Zhang Q, Cao H, . An international collaboration to harmonize the quantitative plasma Epstein-Barr virus DNA assay for future biomarker-guided trials in nasopharyngeal carcinoma. Clin Cancer Res. 2013;19(8):2208-2215. doi:10.1158/1078-0432.CCR-12-3702 23459720 PMC3630245

