# Supplementary material for: Assessment of Survival Model Performance Following Inclusion of Epstein-Barr Virus DNA Status in Conventional TNM Staging Groups in Epstein-Barr Virus–Related Nasopharyngeal Carcinoma
Source: JAMA Netw Open. 2021 Sep 23;4(9):e2124721. doi: 10.1001/jamanetworkopen.2021.24721 (PMC8461502; doi:10.1001/jamanetworkopen.2021.24721)
Supplement: Supplement. — eAppendix. Supplementary Methods eTable. Characteristics of the 2354 Patients with Nonmetastatic Nasopharyngeal Carcinoma Stratified by EBV DNA Status in the Entire Cohort eFigure 1. Unadjusted Kaplan-Meier Survival Analyses of Progression-Free Survival and Overall Survival Stratified by the EBV DNA Status; Inverse Probability Weighting (IPW)-Adjusted Kaplan-Meier Survival Analyses of Progression-Free Survival and Overall Survival Stratified by the EBV DNA Status eFigure 2. Covariate Balances Between Patients With EBV DNA (-) and EBV DNA (+) NPC Assessed Using Standardized Mean Differences (SMDs) eFigure 3. Prognostic Value of EBV DNA Status for Patients With Stage I to IVa in Subgroup Analyses eFigure 4. Kaplan-Meier Survival Analyses for Progression-Free Survival and Overall Survival Stratified by the T Category in Patients With EBV DNA (-) NPC and Patients With EBV DNA (+) NPC eFigure 5. Kaplan-Meier Survival Analyses for Progression-Free Survival and Overall Survival Stratified by the N Category in Patients With EBV DNA (-) NPC and Patients With EBV DNA (+) NPC eFigure 6. Kaplan-Meier Survival Analyses for Progression-Free Survival and Overall Survival Stratified by the 8th Edition of the TNM Staging System in Patients With EBV DNA (-) NPC and Patients With EBV DNA (+) NPC eFigure 7. Optimal Number of Clusters Determined by the Silhouette Method eFigure 8. RPA groups Generated by Hierarchical Clustering eFigure 9. Kaplan-Meier Survival Analyses Stratified by the RPA Stage in the Validation Cohorts eFigure 10. Reclassification of the 8th TNM Stage Using the Proposed RPA staging system eFigure 11. Calibration Plots for Predicting 3- and 5-Year PFS in the Sun Yat-Sen University Cancer Center training cohort, Sun Yat-Sen University Cancer Center Internal Validation Cohort, and Foshan External Validation Cohort [file jamanetwopen-e2124721-s001.pdf]

Supplemental Online Content

Li WZ, Wu HJ, Lv SH, et al. Assessment of survival model performance following inclusion of Epstein-Barr virus DNA status in conventional TNM staging groups in Epstein-Barr virus-related nasopharyngeal carcinoma. *JAMA Netw Open.* 2021;4(9):e2124721. doi:10.1001/jamanetworkopen.2021.24721

eAppendix. Supplementary Methods

eTable. Characteristics of the 2354 Patients with Nonmetastatic Nasopharyngeal Carcinoma Stratified by EBV DNA Status in the Entire Cohort

eFigure 1. Unadjusted Kaplan-Meier Survival Analyses of Progression-Free Survival and Overall Survival Stratified by the EBV DNA Status; Inverse Probability Weighting (IPW)Adjusted Kaplan-Meier Survival Analyses of Progression-Free Survival and Overall Survival Stratified by the EBV DNA Status

eFigure 2. Covariate Balances Between Patients With EBV DNA (-) and EBV DNA (+) NPC Assessed Using Standardized Mean Differences (SMDs)

eFigure 3. Prognostic Value of EBV DNA Status for Patients With Stage I to IVa in Subgroup Analyses

eFigure 4. Kaplan-Meier Survival Analyses for Progression-Free Survival and Overall Survival Stratified by the T Category in Patients With EBV DNA (-) NPC and Patients With EBV DNA (+) NPC

eFigure 5. Kaplan-Meier Survival Analyses for Progression-Free Survival and Overall Survival Stratified by the N Category in Patients With EBV DNA (-) NPC and Patients With EBV DNA (+) NPC

eFigure 6. Kaplan-Meier Survival Analyses for Progression-Free Survival and Overall Survival Stratified by the 8th Edition of the TNM Staging System in Patients With EBV DNA (-) NPC and Patients With EBV DNA (+) NPC

eFigure 7. Optimal Number of Clusters Determined by the Silhouette Method

eFigure 8. RPA groups Generated by Hierarchical Clustering

eFigure 9. Kaplan-Meier Survival Analyses Stratified by the RPA Stage in the Validation Cohorts

eFigure 10. Reclassification of the 8th TNM Stage Using the Proposed RPA staging system

© 2021 Li WZ et al. *JAMA Network Open.*

eFigure 11. Calibration Plots for Predicting 3- and 5-Year PFS in the Sun Yat-Sen University Cancer Center training cohort, Sun Yat-Sen University Cancer Center Internal Validation Cohort, and Foshan External Validation Cohort

This supplemental material has been provided by the authors to give readers additional information about their work.

© 2021 Li WZ et al. *JAMA Network Open.*

eAppendix. Supplementary Methods

Inclusion and Exclusion Criteria of the Study

Patients who fulfilled all the following criteria were enrolled (i) previously untreated nonmetastatic (M0) NPC; (ii) histological subtype of WHO type II or III NPC; (iii) positivity on EBV viral antigen; (iv) treatment with definitive radiotherapy using the intensity-modulated radiotherapy (IMRT) technique with or without platinum-based chemotherapy; and (v) availability of data on baseline plasma EBV DNA titers. Patients with metastatic NPC (M1 disease) at presentation were excluded from developing the refined staging system because they have a poor prognosis regardless of T and N categories and EBV DNA status.

Measurement of EBV DNA Titer

Samples of peripheral blood (3–5 mL) were collected within two weeks before treatment. The blood samples were deposited in an ethylene diaminete traacetic acide tube after collection and centrifuged at 1600g for 15 minutes to separate the plasma and peripheral blood cells. The processed samples were then subjected to DNA extraction within four hours. DNA from plasma samples was extracted using the QIAamp Blood Kit (Qiagen, Hilden, Germany). Plasma EBV DNA titers were measured using the real-time quantitative polymerase-chain-reaction (PCR) assay. The real-time quantitative PCR assay was developed for plasma EBV DNA detection that targets the BamHI-W region. The sequences of the forward and reverse primers used in the PCR assay were 5`-GCCAG AGGTA AGTGG ACTTT-3` and 5`-TACCA CCTCC TCTTC TTGCT-3`. The

© 2021 Li WZ et al. *JAMA Network Open.*

dual fluorescently-labeled oligomer of 5`-(FAM) CACAC CCAGG CACACACTAC ACAT (TAMRA)-3` acted as the probe. More details of experimental procedures for detecting plasma EBV DNA have been described elsewhere [1]. In the current study, EBV DNA (-) was defined as undetectable circulating EBV DNA in pretherapy peripheral blood. EBV DNA (+) was defined as detectable circulating EBV DNA in pretherapy peripheral blood.

Brief Introduction of autoRPA

Recursive Partitioning Analysis (RPA) represents the most recognized approach for cancer prognosis staging. In 1963, Morgan and Sonquist firstly proposed the RPA as a tree-based regression model [2]. Later, Gordon and Olshen extended this algorithm adapting to time-to-event data using log-rank test statistics as splitting criteria in 1985 [3]. RPA can easily handle several prognostic factors in a decision tree model and is the most suitable technique for developing cancer staging schemes. The autoRPA, freely available at http://rpa.renlab.org, provides an online service for building cancer staging models using the RPA algorithm and the log-rank test statistics in an interactive way [4]. One can easily derive a prognostic staging model by pasting the RSD format Files (including survival time, censoring indicator, and user-selected covariates) online. For more details of how to use the autoRPA, please refer to the online Help document.

Details of Inverse Probability Weighting (IPW) Approach

We applied the IPW method to control selection bias to reduce observed differences in baseline characteristics between the two treatment groups [5]. Notably, we defined IPW as the inverse of the probability of EBV DNA status. IPW was calculated based on

© 2021 Li WZ et al. *JAMA Network Open.*

propensity scores estimated from a logistic regression model using a broad set of baseline variables, including age, sex, BMI, smoking, histology, T category, N Stage, clinical-stage, treatment. Here, the IPWs was calculated using the formula: $W_{ATE} = \frac{Z}{e} + \frac{1-Z}{1-e}$. Z is an indicator denoting treatment status (Z = 0 for EBV DNA [-] vs. Z = 1 for EBV DNA [+]), while e represents the propensity score. Using the IPWs, we evaluated the average treatment effect (ATE), which yields a synthetic sample where the observed baseline covariates could not be confounded with treatment assignment. We evaluated covariate balances before and after IPW, using absolute standardized differences (ASD). An ASD of 0.1 or less represented a good balance.

eReferences

1. Shao JY, Li YH, Gao HY, et al. Comparison of plasma Epstein-Barr virus (EBV) DNA levels and serum EBV immunoglobulin A/virus capsid antigen antibody titers in patients with nasopharyngeal carcinoma. Cancer 2004, 100(6):1162-1170.

2. James N. Morgan & John A. Sonquist (1963) Problems in the Analysis of Survey Data, and a Proposal, Journal of the American Statistical Association, 58:302, 415-434, DOI: 10.1080/01621459.1963.10500855.

3. Gordon L, Olshen RA. Tree-structured survival analysis. Cancer Treat Rep1985;69:1065.

4. Yubin Xie, Xiaotong Luo, Huiqin Li, Qingxian Xu, Zhihao He, Qi Zhao*, Zhixiang Zuo*, Jian Ren*. Computational and Structural Biotechnology Journal 18 (2020) 3361–3367.

© 2021 Li WZ et al. *JAMA Network Open.*

5. Austin PC, Stuart EA. Moving towards best practice when using inverse probability of treatment weighting (IPTW) using the propensity score to estimate causal treatment effects in observational studies. Statistics in Medicine. 2015;34:3661-79.

© 2021 Li WZ et al. *JAMA Network Open.*

eTable. Characteristics of the 2354 Patients with Nonmetastatic Nasopharyngeal Carcinoma Stratified by EBV DNA Status in the Entire Cohort

Characteristics	EBV DNA (-) NPC (N=1016, 43.2%)	EBV DNA (+) NPC (N=1338, 56.8%)	*P* value
Age, median [IQR], y	45.0 [38.0-53.0]	45.0 [38.0-53.0]	0.926
Sex			0.395
Female	288 (28.3)	357 (26.7)	
Male	728 (71.7)	981 (73.3)	
BMI (kg/m^2)	21.1 [19.0-23.7]	20.6 [18.9-22.8]	0.001
Smoking			0.021
No	717 (70.6)	883 (66.0)	
Yes	299 (29.4)	455 (34.0)	
Histology			0.084
Type II	31 (3.1)	25 (1.9)	
Type III	985 (96.9)	1313 (98.1)	
Tumor category			<0.001
T1	120 (11.8)	58 (4.3)	
T2	197 (19.4)	161 (12.0)	
T3	496 (48.8)	697 (52.1)	
T4	203 (20.0)	422 (31.5)	
Node category			<0.001
N0	204 (20.1)	110 (8.2)	
N1	419 (41.2)	456 (34.1)	
N2	316 (31.1)	516 (38.6)	
N3	77 (7.6)	256 (19.1)	
Clinical stage			<0.001
I	53 (5.2)	8 (0.60)	
II	151 (14.9)	85 (6.4)	
III	548 (53.9)	637 (47.6)	
IVa	264 (26.0)	608 (45.4)	
Treatment			<0.001
RT alone	181 (17.8)	101 (7.5)	
CRT	835 (82.2)	1237 (92.5)	

© 2021 Li WZ et al. *JAMA Network Open.*

Note: EBV, Epstein-Barr virus; IQR, interquartile range; BMI, body mass index; RT, radiotherapy; CRT, chemoradiotherapy. Data are given as median (interquartile) for continuous variables and frequency (percentage) for categorical variables.

© 2021 Li WZ et al. *JAMA Network Open.*

eFigure 1. Unadjusted Kaplan-Meier Survival Analyses of Progression-Free Survival and Overall Survival Stratified by the EBV DNA Status; Inverse Probability Weighting (IPW)Adjusted Kaplan-Meier Survival Analyses of Progression-Free Survival and Overall Survival Stratified by the EBV DNA Status

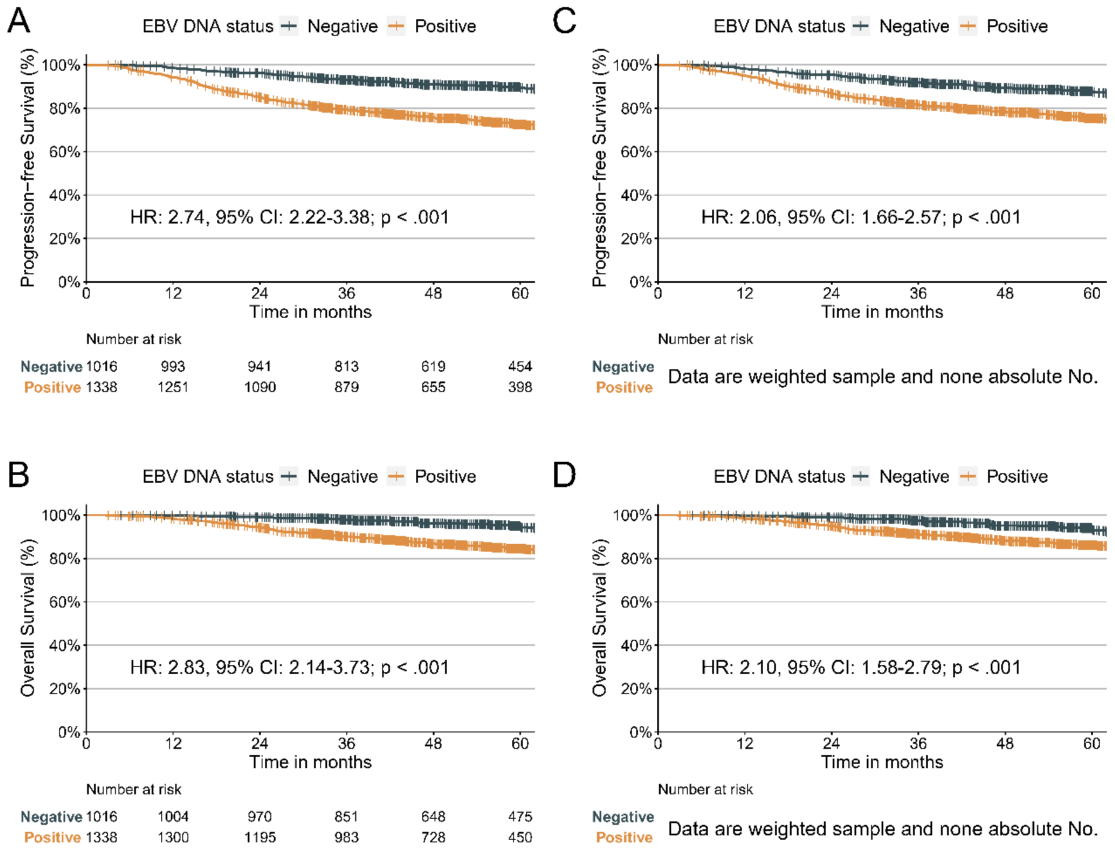

© 2021 Li WZ et al. *JAMA Network Open.*

eFigure 2. Covariate Balances Between Patients With EBV DNA (-) and EBV DNA (+) NPC Assessed Using Standardized Mean Differences (SMDs)

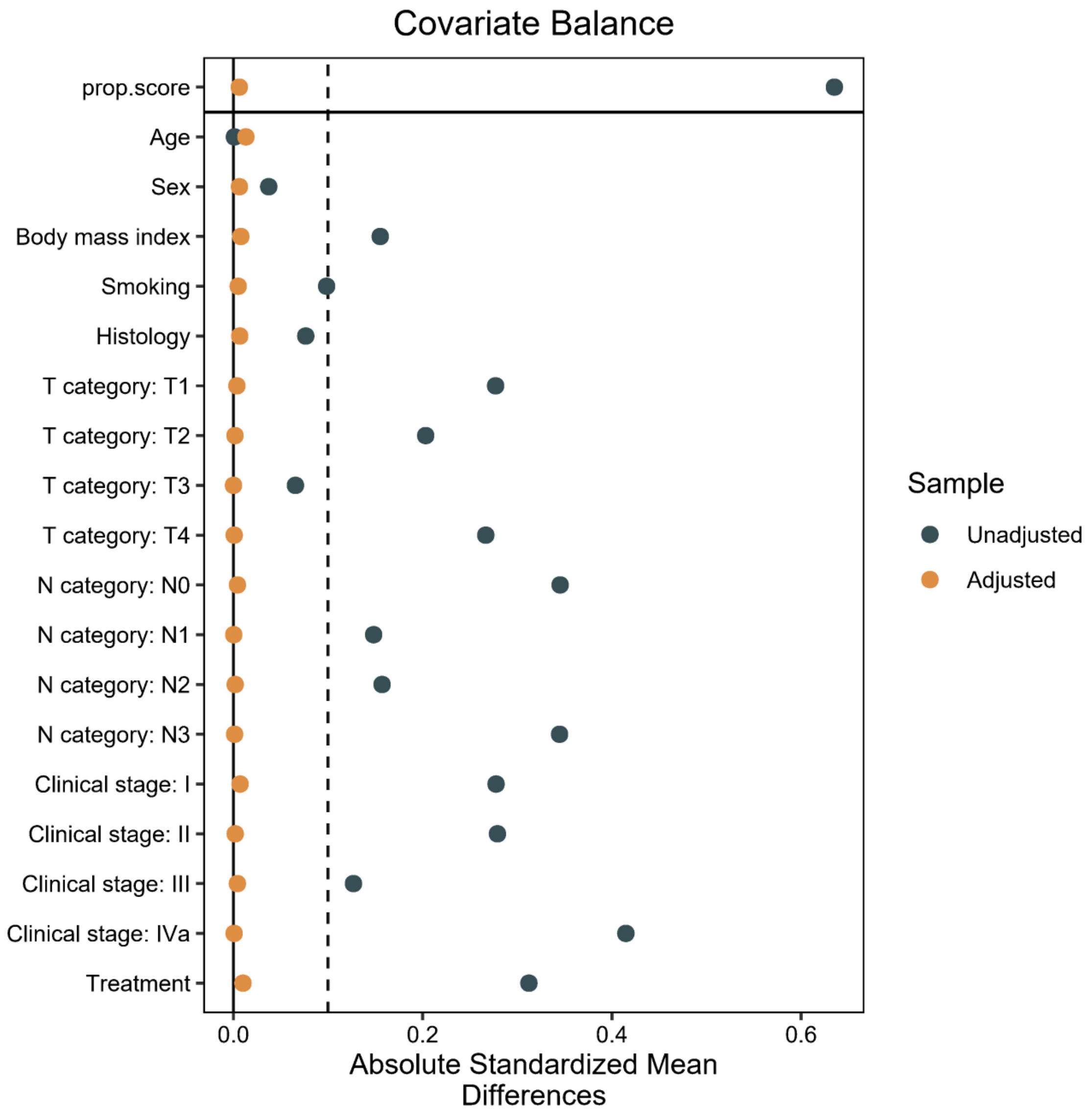

.

© 2021 Li WZ et al. *JAMA Network Open.*

eFigure 3. Prognostic Value of EBV DNA Status for Patients With Stage I to IVa in Subgroup Analyses

Hazard Ratio

Subgroup	No. of Patients (%)	HR (95% CI)	5-y Event Rate EBV DNA (-)	5-y Event Rate EBV DNA (+)	P Value
Progression-free survival					
Total	2354 (100)	2.74 (2.22-3.38)	10.4	27.4	<.001
Clinical stage					
I	61 (2.6)	0 (0-NA)	2	0	.999
II	236 (10)	1.20 (0.48-3.03)	4.1	7	.700
III	1185 (50.3)	2.67 (1.94-3.69)	9	22.9	<.001
IVa	872 (37)	2.06 (1.51-2.80)	19.6	35.5	<.001
Overall survival					
Total	2354 (100)	2.83 (2.14-3.73)	5.4	15.3	<.001
Clinical stage					
I	61 (2.6)	0 (0-NA)	2	0	.999
II	236 (10)	1.10 (0.34-3.61)	2.2	3.2	.871
III	1185 (50.3)	2.84 (1.86-4.33)	5.4	13.1	<.001
IVa	872 (37)	2.23 (1.47-3.38)	8.8	19.8	<.001

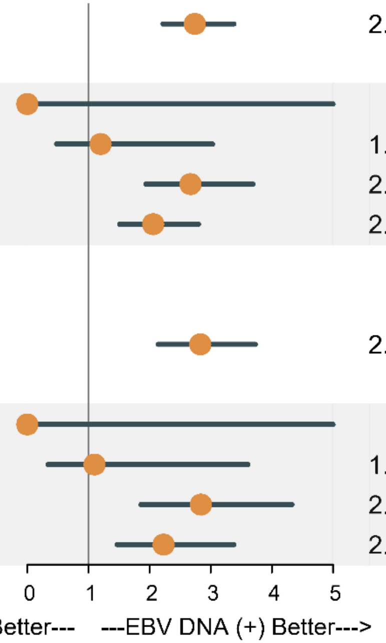

© 2021 Li WZ et al. *JAMA Network Open.*

eFigure 4. Kaplan-Meier Survival Analyses for Progression-Free Survival and Overall Survival Stratified by the T Category in Patients With EBV DNA (-) NPC and Patients With EBV DNA (+) NPC

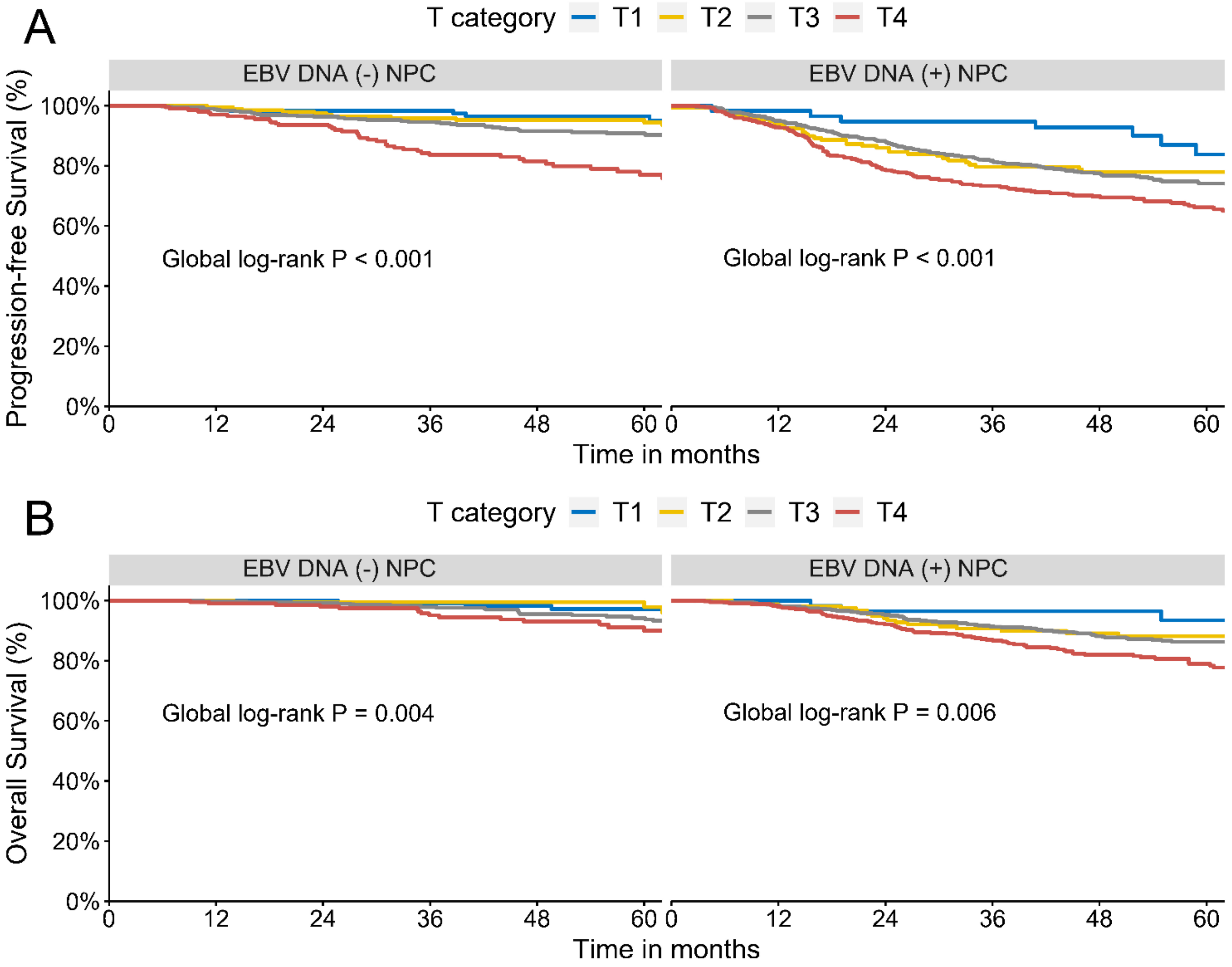

© 2021 Li WZ et al. *JAMA Network Open.*

eFigure 5. Kaplan-Meier Survival Analyses for Progression-Free Survival and Overall Survival Stratified by the N Category in Patients With EBV DNA (-) NPC and Patients With EBV DNA (+) NPC

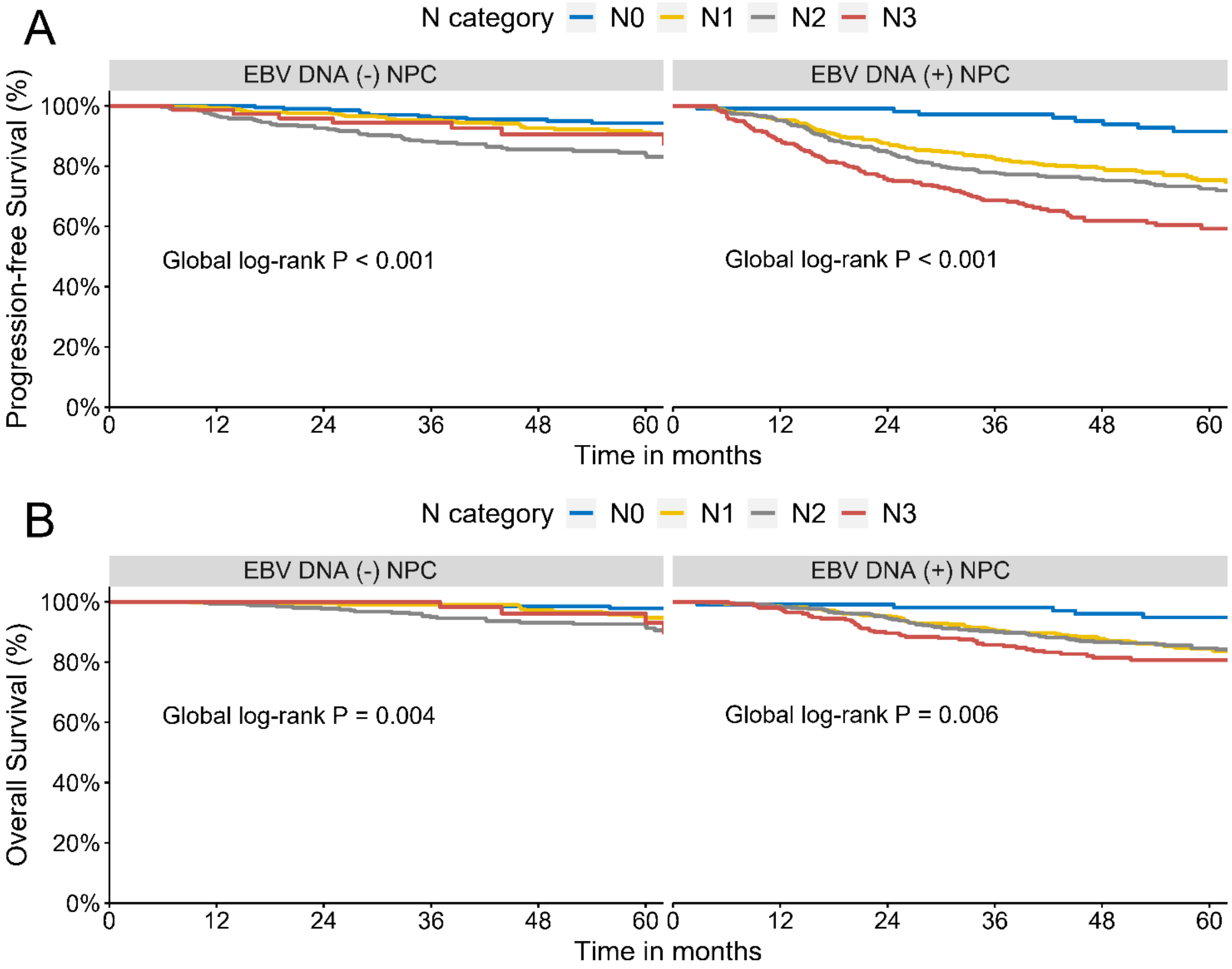

© 2021 Li WZ et al. *JAMA Network Open.*

eFigure 6. Kaplan-Meier Survival Analyses for Progression-Free Survival and Overall Survival Stratified by the 8th Edition of the TNM Staging System in Patients With EBV DNA (-) NPC and Patients With EBV DNA (+) NPC

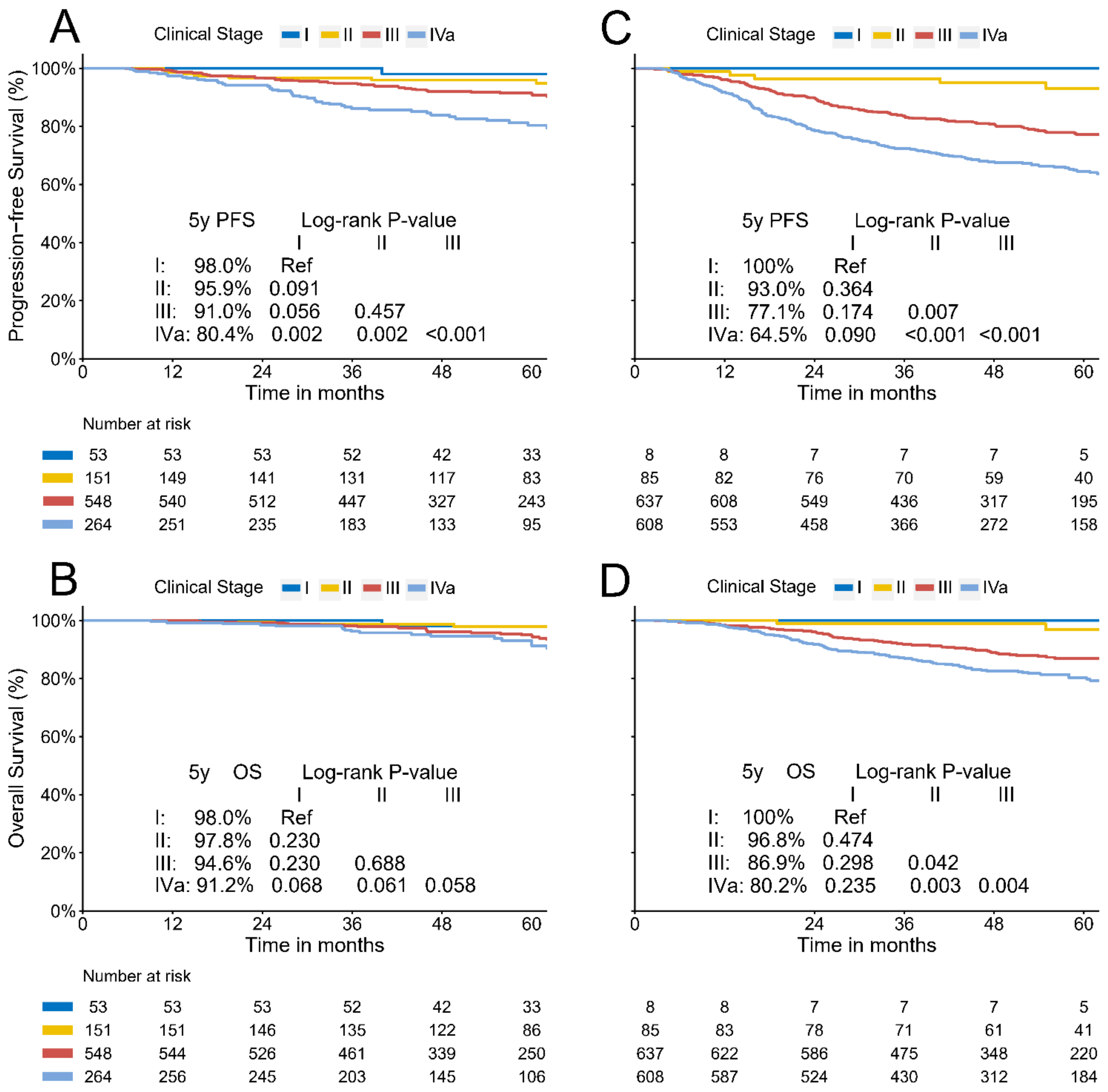

© 2021 Li WZ et al. *JAMA Network Open.*

eFigure 7. Optimal Number of Clusters Determined by the Silhouette Method

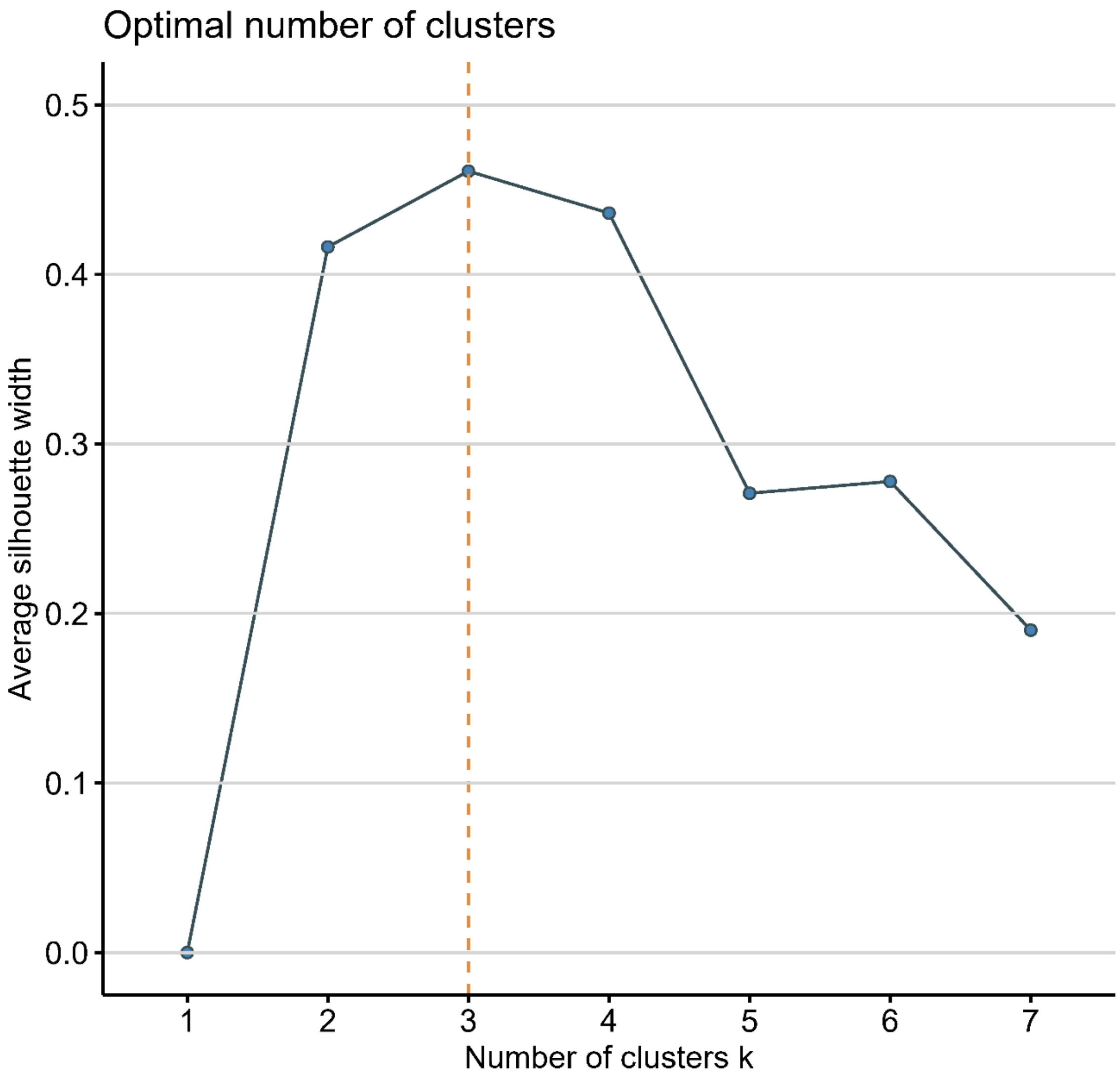

© 2021 Li WZ et al. *JAMA Network Open.*

eFigure 8. RPA groups Generated by Hierarchical Clustering

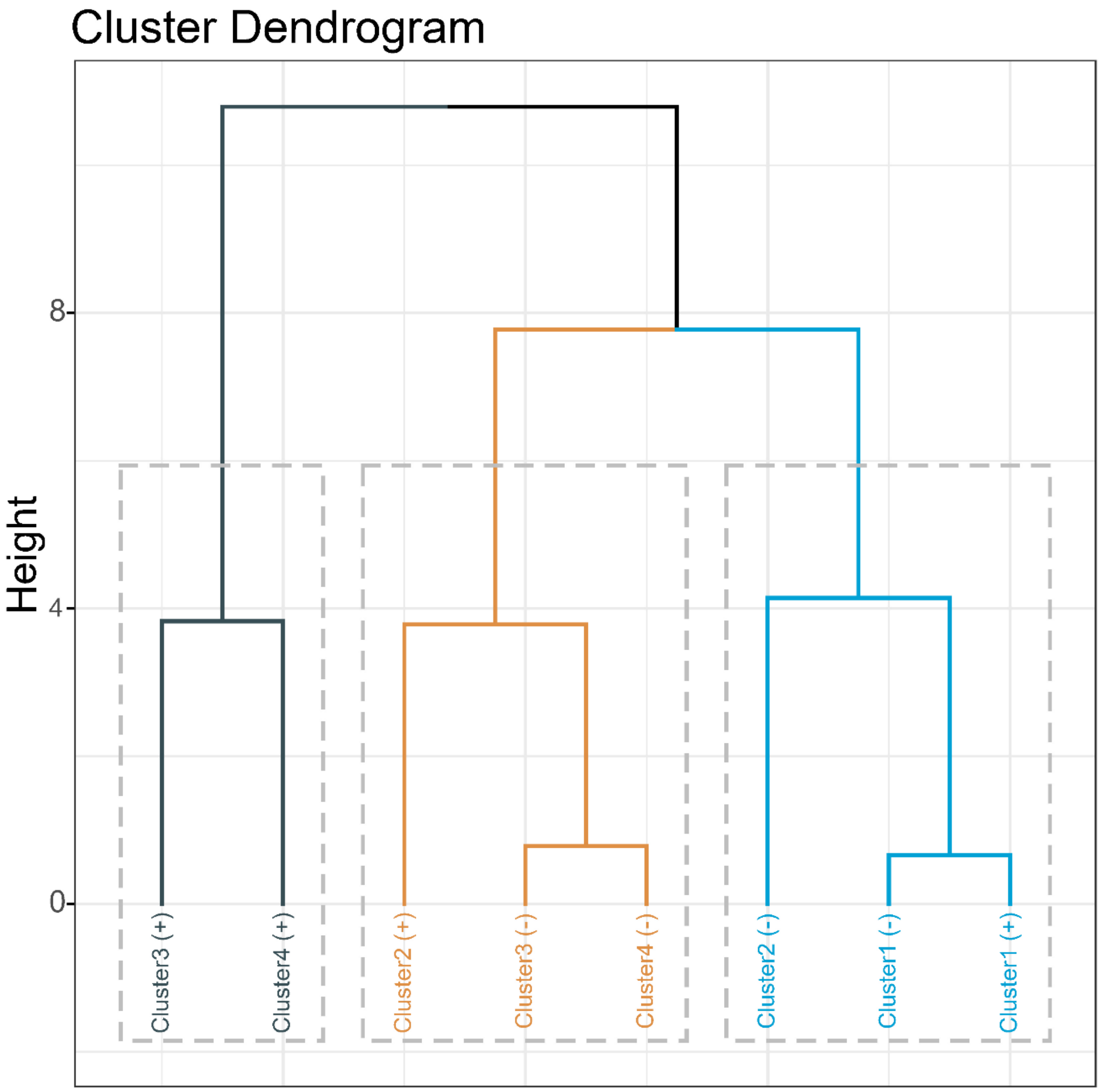

© 2021 Li WZ et al. *JAMA Network Open.*

eFigure 9. Kaplan-Meier Survival Analyses Stratified by the RPA Stage in the Validation Cohorts

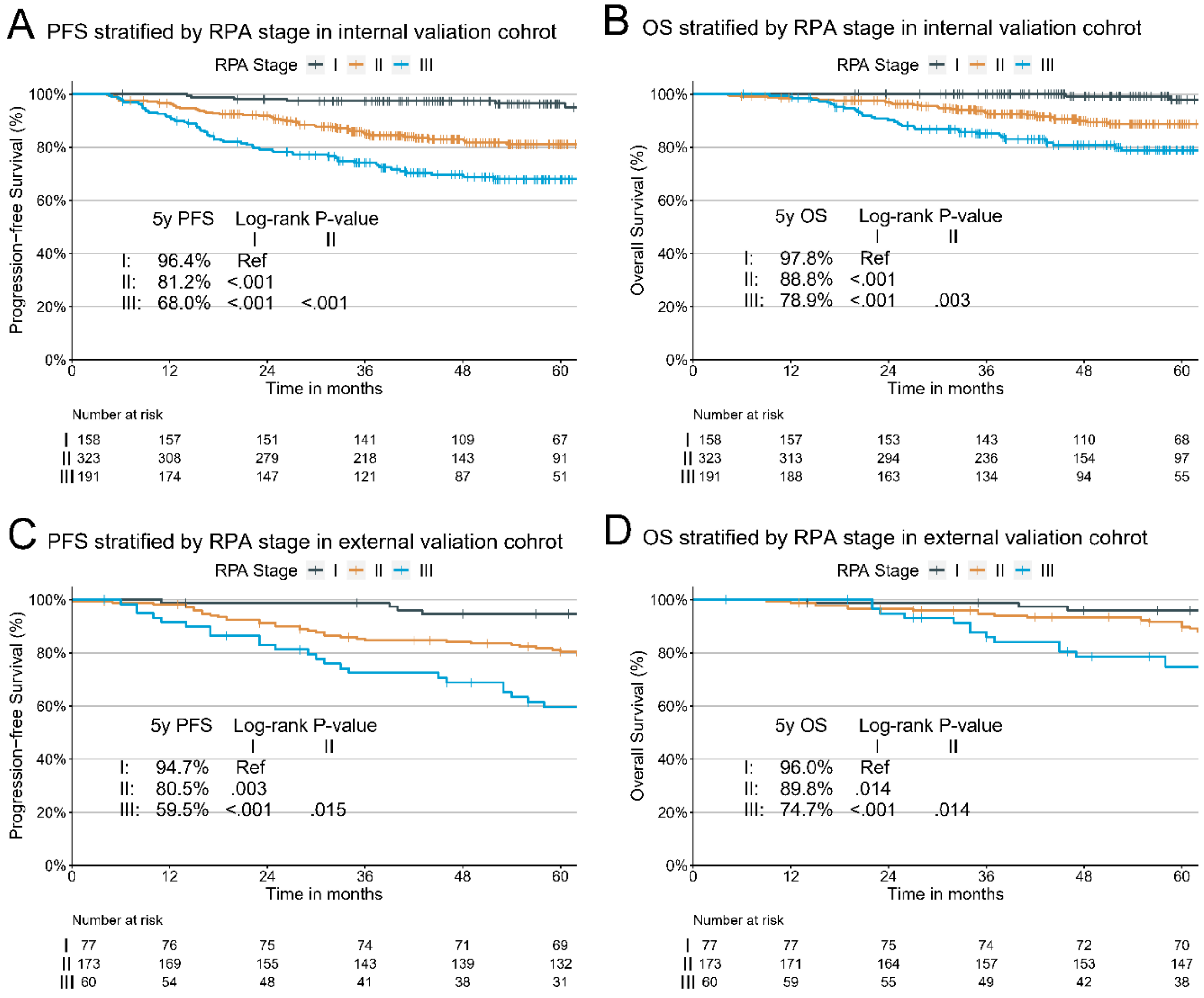

© 2021 Li WZ et al. *JAMA Network Open.*

eFigure 10. Reclassification of the 8th TNM Stage Using the Proposed RPA staging system

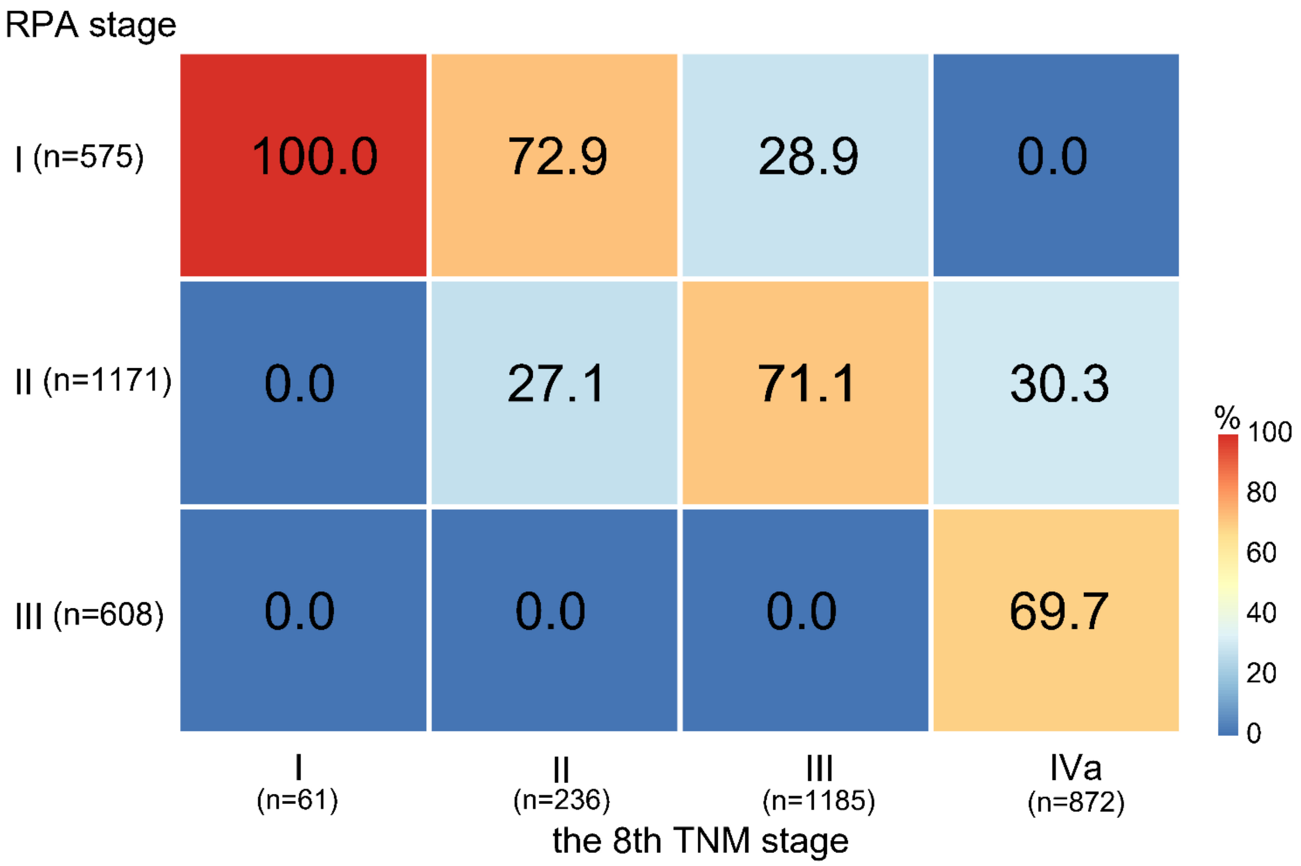

© 2021 Li WZ et al. *JAMA Network Open.*

eFigure 11. Calibration Plots for Predicting 3- and 5-Year PFS in the Sun Yat-Sen University Cancer Center training cohort, Sun Yat-Sen University Cancer Center Internal Validation Cohort, and Foshan External Validation Cohort

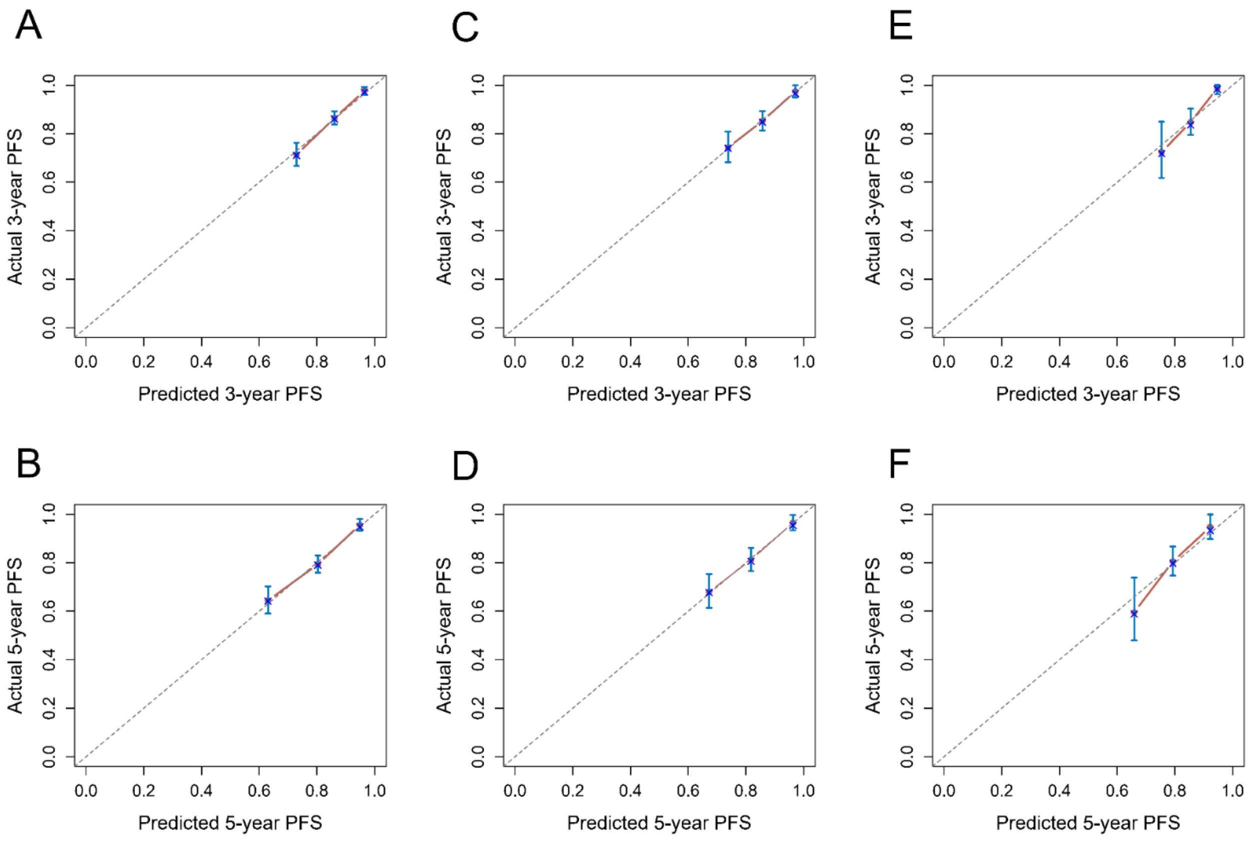

© 2021 Li WZ et al. *JAMA Network Open.*
